# Self-care behaviours among people with type 2 diabetes mellitus in South Asia: A systematic review and meta-analysis

**DOI:** 10.7189/jogh.12.04056

**Published:** 2022-08-03

**Authors:** Grish Paudel, Corneel Vandelanotte, Padam K Dahal, Tuhin Biswas, Uday N Yadav, Tomohiko Sugishita, Lal Rawal

**Affiliations:** 1 School of Health, Medical and Applied Sciences, Central Queensland University, Sydney, Australia; 2 Appleton Institute, Physical Activity Research Group, Central Queensland University, Rockhampton, Australia; 3 The Australian Research Council Centre of Excellence for Children and Families over the Life Course, The University of Queensland, Brisbane, Queensland, Australia; 4 National Centre for Epidemiology and Population Health, The Australian National University, Canberra, Australia; 5 Centre for Primary Health Care and Equity, University of New South Wales, Sydney, Australia; 6 Section of Global Health, Division of Public Health, Department of Public Health, Tokyo Women's Medical University, Tokyo, Japan; 7 Translational Health Research Institute (THRI), Western Sydney University, Sydney Australia

## Abstract

**Background:**

The burden of Type 2 Diabetes Mellitus (T2DM) in South Asian countries is increasing rapidly. Self-care behaviour plays a vital role in managing T2DM and preventing complications. Research on self-care behaviours among people with T2DM has been widely conducted in South Asian countries, but there are no systematic reviews that assess self-care behaviour among people with T2DM in South Asia. This study systematically assessed the studies reporting self-care behaviours among people with T2DM in South-Asia.

**Methods:**

Adhering to the PRISMA guidelines, we searched six bibliographic databases (Scopus, PubMed, CINAHL, Embase, Web of Science, and PsychInfo) to identify the relevant articles published between January 2000 through March 2022. Eligibility criteria included all observational and cross-sectional studies reporting on the prevalence of self-care behaviours (ie, diet, physical activity, medication adherence, blood glucose monitoring, and foot care) conducted in South Asian countries among people with T2DM.

**Results:**

The database search returned 1567 articles. After deduplication (n = 758) and review of full-text articles (n = 192), 92 studies met inclusion criteria and were included. Forward and backward reference checks were performed on included studies, which resulted in an additional 18 articles. The pooled prevalence of adherence to blood glucose monitoring was 65% (95% CI = 49-80); 64% for medication adherence (95% CI = 53-74); 53% for physical activity (95% CI = 39-66); 48% for diet (95% CI = 38-58); 42% for foot care (95% CI = 30-54). About a quarter of people with T2DM consumed alcohol (25.2%, IQR = 13.8%-38.1%) and were using tobacco products (18.6%, IQR = 10.6%-23.8%).

**Conclusions:**

Our findings suggest that the prevalence of self-care behaviours among people with T2DM in South Asia is low. This shows an urgent need to thoroughly investigate the barriers to the practising of self-care and design and implement interventions to improve diabetes self-care behaviour among people with T2DM in South Asia.

Diabetes mellitus is a major public health concern worldwide. The number of people with diabetes mellitus has increased from 108 million in 1980 to 422 million in 2014 [1] and 537 million in 2021 [2]. According to the International Diabetes Federation (IDF), this number is likely to reach 643 million by 2030 [2]. Type 2 Diabetes Mellitus (T2DM) constitutes more than 90% of all diabetes cases around the globe [2]. In recent years, the T2DM prevalence has significantly increased in low- and middle-income countries compared to higher-income countries in recent years [3]. The prevalence of T2DM in the South-Asian region specifically has doubled from 4.1% in 1980 to 8.6% in 2014 [3] and is estimated to reach 11.3% by 2045 [2].

South Asia is the southern region of Asia that comprises eight countries: Nepal, India, Bangladesh, Maldives, Sri Lanka, Pakistan, Bhutan, and Afghanistan [4]. South Asians are at higher risk of developing Non-Communicable Disease (NCDs), including T2DM, compared to other ethnic groups [5]. They tend to have more abdominal fat, more insulin resistance, low levels of adiponectin, low high-density lipoproteins, high low-density lipoproteins, and high triglycerides – characteristics which are responsible for the development of T2DM and cardiovascular diseases [5]. The prevalence of T2DM is the highest in Pakistan (26.7%) followed by India (8.3%), Bhutan (8.8%), Sri Lanka (9.8%), Bangladesh (12.5%), Maldives (6.7%), Afghanistan (8.7%) and Nepal (6.3%) [2]. The increased prevalence of T2DM negatively affects the socioeconomic circumstances for South Asian people by increasing diabetes-related health expenditure [6]. Poor knowledge about the disease, delayed diagnosis, poor adherence to self-care behaviours, and administration of harmful alternative medicines are the challenges for the treatment of T2DM among South-Asians [7,8].

The IDF has identified indicators for data collection (at least once in 12-24 months) to monitor the effectiveness of diabetes management, including self-care. The components of self-care are smoking status, alcohol consumption, self-monitoring (glucose, blood pressure, body weight), diet, physical activity, driving risk, medication adherence, insulin techniques, and dental care [9]. Similarly, the American Association of Diabetes Educators (AADE) has identified seven self-care behaviours: healthy eating, being active, monitoring, taking medication, problem-solving, healthy coping, and reducing risks as a framework for delivering patient-oriented diabetes care and education [10]. Adherence to self-care behaviours is essential for controlling adequate metabolism and preventing long-term complications [9,11-13]. Adherence to healthier behaviours significantly reduces the T2DM related complications and the mortality rate [14,15]. Despite this evidence, South-Asians adhere poorly to T2DM self-care behaviours [7,8,16]. To date, the components of self-care for management of T2DM in the South Asian region have not been defined by South Asian or the regional federations on diabetes.

As many South Asian countries have adopted the WHO’s Global Action Plan for the Prevention and Control of Non-Communicable Diseases [17], there is a need for research examining the planning, implementation, and evaluation of NCD prevention, control, and management strategies. Similarly, the higher risks of developing T2DM among South Asians have drawn the attention of policy makers in management and control of T2DM in this region. There are many studies examining the prevalence of self-care behaviours among those with T2DM in South-Asian countries [18-21]. However, a comprehensive systematic review and meta-analysis of this collective body of literature has not yet been conducted; posing challenges for policymakers to act on this. The literature on the prevalence of self-care behaviours among people with T2DM has been systematically analysed in regions other than South Asia [11,22-25] and reported widely varying rates of self-care behaviours. The findings from such reviews improve our understanding on practice of self-care among the people with T2DM across the regions. Because of the increasing burden of T2DM in South Asia [2] and South Asians being at higher risk for developing T2DM [5], there is an urgent need to design and implement effective prevention and management programs for T2DM. Improving our understanding on the practice of self-care behaviours among people with T2DM will help forward this agenda. This systematic review and meta-analysis aim to assess and summarize the findings on self-care behaviours among people with T2DM in South-Asia.

## METHODS

This systematic review is reported in accordance with the Preferred Reporting Items for Systematic Reviews and Meta-Analysis (PRISMA) guidelines [26]. The South Asian countries included in this study were Bangladesh, Bhutan, India, Nepal, Pakistan, Maldives, Sri Lanka, and Afghanistan [4]. The review was registered with PROSPERO, an international prospective register of systematic reviews (Registration number: CRD42021242930).

### Search strategy

We systematically searched six bibliographic databases: Scopus, PubMed, CINAHL, Embase, Web of Science, and PsychInfo for articles published between January 2000 through March 2022. This time frame aligns with the launch of the Millennium Development Goals (MDGs) in 2000 [27]. A search strategy for each database was developed with all the possible combinations of three keywords, “Type 2 Diabetes Mellitus”, “Self-care behaviour”, and “South-Asia” (See Appendix S1 in the **Online Supplementary Document**). Search tools such as PICO, PICOS, or SPIDER were not used because this study only reviewed observational and cross-sectional studies. Medical Subject Headings (MeSH), boolean operators, wildcards, truncation, and field tags were used where appropriate. Both the reference lists (ie, backward search) and articles citing (ie, forwards search) of included studies were checked by two authors (GP, PD) for additional relevant studies.

### Inclusion and exclusion criteria

Observational, and cross-sectional studies that quantitatively reported on the practice of self-care behaviours (ie, diet, physical activity, medication adherence, blood glucose monitoring, and foot care) among adults with T2DM from South-Asian countries that were published in the English language were included. These five self-care behaviours were based on the key indicators for self-care behaviour as suggested by the IDF and AADE [9,10]. Furthermore, these domains of self-care were also assessed by several review studies on self-care among people with T2DM in other settings [11,22-25]. Studies not mentioning the type of diabetes examined, studies based on the same data set, and studies without a full-text publication available were excluded.

### Screening

**Figure 1:**
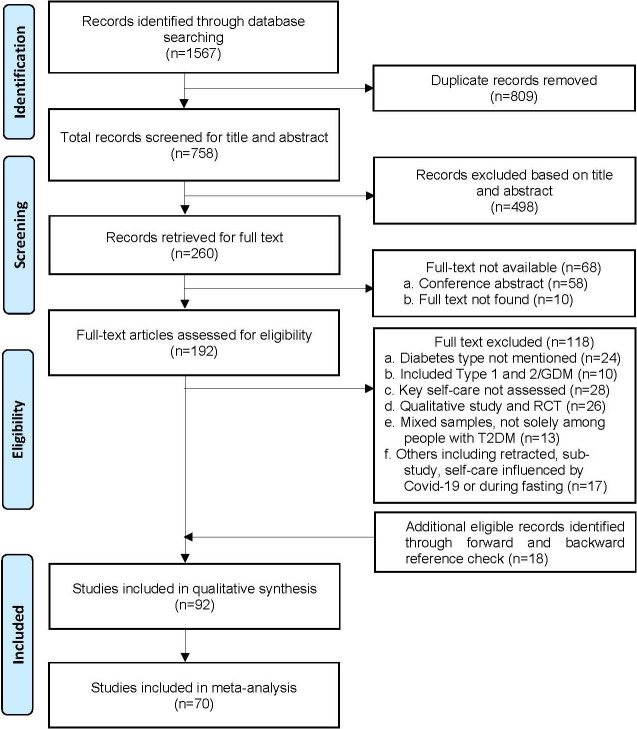
PRISMA flow diagram (2009) for reporting systematic review and meta-analysis.

One author (GP) performed the online database search in first week of April 2021 (updated on 25 March 2022). Articles identified through the search were exported into the EndNote referencing software and deduplicated. Titles and abstracts were screened independently by two reviewers (GP, PD). The potentially eligible studies underwent full-text screening using the selection criteria. Disagreements between the two reviewers (GP and PD) was discussed in consultation with a third reviewer (LR). Remaining disagreements were discussed within the study team (GP, PD, TB, LR, UNY, TS, and CV) until a consensus was reached. A detailed study selection process is presented in the PRISMA flowchart (**Figure 1**) [26].

### Data extraction and quality assessment

A data extraction template similar to the one used in the systematic review of Stephani et al. [24] was developed in Microsoft Excel to collect information from the selected studies for the analysis. Information on the primary author, publication year, country, study design, sample size, demographic characteristics of the population (eg, age, gender, and other contextual information), and reported self-care behaviours were extracted.

The Jonna Briggs Institute (JBI) critical appraisal checklist was used to assess the methodological quality of the selected studies following each study design [28]. Two independent reviewers (GP, PD) critically appraised the selected articles using the JBI critical appraisal checklist. This tool involves assessing the study’s methodological quality in dealing with bias at different study stages. A checklist for analytical cross-sectional studies (with 8 appraising items) and another for prevalence studies (with 9 appraising items) were used. Each response was scored with 1 (if the response to the question was “yes”) and 0 (if the response to the question was “no” or “unclear” or “not applicable”). Based on the score, studies were categorized into high (80% and above), moderate (60%-80%) and low quality (<60%) [29]. Discrepancies between reviewers (GP, PD) on study quality assessment were resolved through discussion and consultation with a third reviewer (LR).

### Meta-analysis

A systematic narrative synthesis was performed to describe the characteristics and results of all included studies. The narrative synthesis followed the Guidance of the Conduct of Narrative Synthesis in Systematic Reviews [30]. Data synthesis and analysis were performed by one reviewer (GP) and the findings were discussed with all other team members.

In the meta-analysis, the overall pooled prevalence for each domain of self-care behaviours was conducted. A subgroup analysis of self-care behaviours including diet, physical activity, and foot care was conducted based on studies that either used a standardised tool to assess self-care domains or those that clearly defined the self-care domain and studies that did not clarify either the measure used to assess self-care domain or that did not define self-care domain. Medication adherence was not considered for subgroup analysis as most included studies used standard tools, while some reported regular intake of medication as recommended by their health care providers. Similarly, blood glucose monitoring differs from person to person based on their blood glucose level, so subgroup analysis was based on studies that assessed blood glucose monitoring on monthly basis and studies that assessed blood glucose monitoring at least once in three months. We used the quality effects model (QE) for bias adjustment [31]. The advantage of the QE model is that the between-study variability is adjusted based on the relative quality rank of the studies instead of on random variables assigned by the random effect (RE) model. The heterogeneity of the studies was reported by the I-squared value (I^2^) which measures the proportion of total variance between studies beyond random error [32]. As significant heterogeneity was detected among the studies (*I*^2^>50%) in the meta-analysis, a random-effects model was used. All the analyses were conducted using the MetaXL software version 5.3 [33]. Publication bias was assessed using both a graphical (Doi plot) and quantitative (Luis Furuya-Kanamori (LFK) index) examination for potential small-study effects [32]. LFK indices are defined as no (±1), minor (between ±1 and ±2), and major (>±2) asymmetry, respectively. Sensitivity and subgroup analysis were performed for extreme levels of heterogeneity between studies (*I*^2^≥90%) [32].

## RESULTS

### Study selection

A total of 1585 studies were identified through the database search (n = 1567) and forward and backward reference checking (n = 18). The duplicates (n = 809) were removed, and 758 studies underwent the title and abstract screening. Of these, 260 studies were eligible for full-text retrieval and 192 studies were retrieved with full-text articles. It was not possible to access the full-text of 68 studies, which were conference abstracts or articles published in local paper-based journals. After full text review, 92 studies met the inclusion criteria and were included in the qualitative synthesis, and 70 were eligible to be included in the meta-analysis (22 were excluded due to insufficiently disaggregated data). A detailed process of study screening and selection is presented in the PRISMA flow diagram **(****Figure 1****)**.

### Quality assessment of the included studies

Quality assessment of 92 studies included in this review was done using Jonna Briggs Institute (JBI) Critical Appraisal Tool based on study design. Thirty-five studies (38%) were assessed as high quality, 33 studies (36%) were assessed as moderate quality, and 24 studies (26%) were assessed as poor quality (Appendix S2 in the **Online Supplementary Document**). The detailed information on quality assessment of individual study is presented in **Table 1****.**

**Table 1:** General characteristics of included studies

			Sample characteristics				Reported self-care behaviours					Quality score
**Author**	**Year**	**Country**	**Sample size**	**Male**	**Female**	**Mean age (SD)**	**Diet**	**Physical Activity**	**Medication intake**	**Foot care**	**SMBG**	
Shah, Kamdar and Shah [34]	2009	India	238	120	118	55.8 (±10.2)	X			X	X	Low
Sultana et al. [35]	2010	India	218	104	114	51.5 (±12.3)			X			Moderate
Malathy et al. [36]	2011	India	207	85	122	52.1	X	X	X			Low
Gopichandran et al. [37]	2012	India	200	82	118	NR	X	X	X		X	High
Patel et al. [38]	2012	India	399	259	140	53.1 (±7.9)	X	X			X	High
Sasi et al. [39]	2013	India	546	303	243	55.4	X	X	X	X		Moderate
Arulmozhi and Mahalakshmy [40]	2014	India	150	75	75	54.0 (±12.0)	X	X	X	X		Moderate
Khan et al. [41]	2014	India	184	81	103	51.4 (±12.2)			X			Moderate
Santhanakrishnan, Lakshminarayanan and Kar [42]	2014	India	135	27	108	59.0 (±12.0)	X	X	X	X		Low
Saurabh et al. [43]	2014	India	103	48	55	54.8 (±11.8)	X	X		X	X	Low
Sajith et al. [44]	2014	India	105	60	45	NR	X	X	X			Low
Abraham et al. [45]	2015	India	60	25	35	50.7 (±7.0)	X	X		X	X	High
Divya and Nadig [46]	2015	India	150	104	46	49.1			X			Low
Basu et al. [47]	2015	India	385	159	226	53.1 (±10.2)	X	X	X			High
Rajasekharan et al. [48]	2015	India	290	174	116	47.9 (±8.9)	X	X	X	X	X	High
Das et al. [49]	2016	India	232	199	33	57.0 (±8.9)	X	X	X	X	X	High
Karthikeyan, Madhusudhan and Selvamuthukumaran [50]	2016	India	345	185	160	NR			X			Low
Pathania et al. [51]	2016	India	48	25	23	57.4 (±10.6)			X			Moderate
Dinesh, Kulkarni and Gangadhar [52]	2016	India	400	245	155	NR	X	X	X	X	X	High
Debnath et al. [53]	2017	India	450	253	197	64.8 (±4.6)		X	X	X	X	Moderate
Kumar et al. [54]	2017	India	124	68	56	Median = 60 (IQR = 50-68) years			X		X	High
Samu, Amirthalingam and Mohammed [55]	2017	India	86	38	48	NR			X			High
Sheeba, Ak and Biju [56]	2017	India	100	60	40	NR	X	X	X	X	X	Low
Srinath, Basavegowda and Tharuni [57]	2017	India	400	172	228	NR	X	X	X	X	X	Moderate
Britto et al. [58]	2018	India	25	NR	NR	58.8 (±8.9)		X				Moderate
Pati et al. [59]	2018	India	321	204	117	51.0 (±12.8)	X	X				Low
Ravi, Kumar and Gopichandran [60]	2018	India	200	96	104	NR	X	X		X	X	High
Venkatesan, Dongre and Ganapathy [61]	2018	India	328	149	179	57.3 (±12.1)			X			High
Jasmine and Iyer [62]	2019	India	77	33	44	NR	X	X	X	X	X	Low
Acharya et al. [63]	2019	India	200	74	126	49.8 (±10.5)			X			Low
Aravind, Joy and Rakesh [64]	2019	India	68	39	29	62.5 (±11.2)	X	X			X	Moderate
Banerjee et al. [65]	2019	India	347	210	137	NR		X				Moderate
Raj, Selvaraj and Thomas [66]	2019	India	205	110	95	62.3 (±9.3)		X				High
Sirari et al. [67]	2019	India	60	30	30	54.9 (±9.2)	X	X		X	X	High
Bashir et al. [68]	2020	India	203	99	104	53.9 (±10.5)	X	X				Moderate
Chandrika et al. [69]	2020	India	208	95	113	51.3 (±9.4)	X	X	X		X	High
Kowsalya et al. [70]	2020	India	60	32	28	NR			X			Low
Palathingal et al. [71]	2020	India	200	123	77	NR			X		X	Low
Patnaik et al. [72]	2020	India	100	58	42	54.2 (±12.0)	X					Moderate
Shrivastva et al. [73]	2020	India	166	109	57	NR	X	X			X	Moderate
Achappa [74]	2020	India	70	28	42	58.9 (±14.5)			X			Low
Karthik et al. [75]	2020	India	250	137	113	NR	X	X	X	X	X	Moderate
Kumar et al. [76]	2021	India	105	43	62	54.8 (±8.9)	X	X			X	High
Rana et al. [77]	2021	India	200	100	100	56.2 (±8.3)	X	X	X		X	Low
Verma, et al. [18]	2021	India	416	243	173	NR		X		X		High
Burman et al. [78]	2021	India	367	172	195	51.4 (±9.3)	X	X	X	X	X	Moderate
Durai et al. [79]	2021	India	390	104	286	56.2 (±10.4)	X	X	X	X	X	Moderate
Mishra et al. [80]	2021	India	277	158	119	50.8 (±10.6)			X			Moderate
Singh et al. [81]	2021	India	350	179	171	NR			X			Moderate
Aravindakshan et al. [82]	2021	India	218	87	131	62.1 (±12.2)			X			Moderate
Zuberi, Syed and Bhatti [83]	2011	Pakistan	286	128	158	NR	X	X	X	X		High
Ahmed et al. [84]	2015	Pakistan	139	60	79	43.0 (±16.0)	X	X	X	X	X	Moderate
Javaid et al. [85]	2016	Pakistan	120	38	62	50.7 (±10.6)	X	X				Low
Bukhsh et al. [86]	2017	Pakistan	130	55	75	51.3 (±10.4)	X	X			X	Moderate
Iqbal et al. [87]	2017	Pakistan	300	180	120	51.2 (±9.5)			X			High
Nazirl et al. [88]	2017	Pakistan	392	222	170	50.7 (±9.6)			X			High
Rana et al. [89]	2017	Pakistan	145	54	91	50.2 (±8.5)			X			Low
Bukhsh et al. [90]	2018	Pakistan	218	112	106	50.7 (±13.3)	X	X			X	High
Farooq et al. [91]	2018	Pakistan	180	82	98	50.3 (±11.2)	X					Low
Zafar et al. [92]	2018	Pakistan	220	93	127	52.9 (±12.5)				X		Moderate
Hussain, Said and Khan [93]	2020	Pakistan	524	0	524	64.0			X			Moderate
Siddique et al. [94]	2020	Pakistan	154	68	86	NR	X	X	X		X	Moderate
Malik et al. [19]	2020	Pakistan	363	241	122	45.7	X	X	X	X	X	Moderate
Sayeed et al. [95]	2020	Pakistan	317	174	143	NR	X	X			X	Moderate
Ishaq et al. [96]	2021	Pakistan	300	180	120	51.2 (±9.6)			X			Moderate
Shrestha et al. [97]	2013	Nepal	100	48	52	58.1 (±11.6)			X			Low
Parajuli et al. [98]	2014	Nepal	385	187	198	54.4 (±11.5)	X	X				High
Sharma and Bhandari [99]	2014	Nepal	100	56	44	NR	X	X			X	Low
Bhandari and Kim [20]	2016	Nepal	230	91	139	56.9 (±10.8)	X	X	X	X	X	High
Ghimire [100]	2017	Nepal	197	111	86	54.7 (±11.3)	X	X				High
Shrestha et al. [101]	2017	Nepal	183	116	67	58.7 (±12.9)	X	X	X			Low
Ghimire and Devi [102]	2018	Nepal	115	62	53	60.0 (±10.3)	X	X			X	Moderate
Kadariya and Aro [103]	2018	Nepal	270	167	103	53 (ranging from 30-70 y)		X				High
Sapkota et al. [104]	2018	Nepal	200	116	84	51.9 (±11.5)		X	X		X	Moderate
Thapa [105]	2018	Nepal	141	71	70	NR	X	X	X	X	X	Low
Pokhrel et al. [106]	2019	Nepal	480	236	244	58.3 (±12.5)	X	X	X		X	High
Bhattarai et al. [107]	2019	Nepal	214	104	110	NR	X	X	X		X	Low
Sharma et al. [108]	2021	Nepal	296	120	176	59.5 (±11.7)			X			High
Shrestha et al. [109]	2021	Nepal	354	156	198	51.7 (±12.6)	X	X	X	X	X	High
Kandel et al. [110]	2022	Nepal	411	177	234	NR	X	X	X	X	X	High
Saleh et al. [111]	2012	Bangladesh	160	72	88	45.1 (±5.6)	X	X				Low
Mumu et al. [112]	2014	Bangladesh	374	157	217	51.0 (±11.3)	X	X		X	X	Moderate
Saleh et al. [113]	2014	Bangladesh	500	249	251	54.2 (±11.2)	X	X	X	X	X	High
Ahmed et al. [114]	2017	Bangladesh	122	67	55	57.5 (±8.7)			X		X	High
Chowdhury et al. [115]	2018	Bangladesh	11917	4418	7499	50.0 (±12.0)	X	X			X	Moderate
Bukht et al. [116]	2019	Bangladesh	977	468	509	56.0 (±8.0)		X				High
Majid et al. [117]	2019	Bangladesh	420	248	172	47.2 (±6.4)	X					High
Islam et al. [118]	2020	Bangladesh	265	133	132	50.3 (±9.9)		X		X	X	High
MahmudulHasan et al. [119]	2021	Bangladesh	379	175	204	NR	X	X				High
Mannan et al. [21]	2021	Bangladesh	2061	1233	828	50.6 (±12.1)	X		X			High
Medagama and Galgomuwa [120]	2018	Sri-Lanka	400	113	287	55.4 (±8.9)		X				Moderate
Rathish et al. [121]	2019	Sri-Lanka	200	100	100	NR			X			Moderate

SMBG – self-monitoring of blood glucose, NR – not reported, IQR – interquartile range, SD – standard deviation

### Characteristics of the included studies

Of the total included studies (n = 92), 50 were conducted in India [18,34-82], 15 in Pakistan [19,83-96], 15 in Nepal [20,97-110], 10 in Bangladesh [21,111-119] and two in Sri-Lanka [120,121]. No studies were conducted in the Maldives, Bhutan, and Afghanistan. 78 studies were based on data recorded in health facilities (hospitals, primary health care centres, diabetic clinics, and pharmacies) while 14 studies were based on data collected in community settings. The general characteristics of the studies are summarized in **Table 1**. The total number of participants in the included studies was 36 180 (16 601 male and 19 559 female) and ranged from 48 participants in the smallest study [51] to 11 917 in the largest study [115]. The mean age of the participants, as reported by 66 studies, ranged from 43 to 64 years. 24 studies reported the length of time participants were living with T2DM, which ranged from 1.5 to 9.7 years. Thirty studies reported on smoking habits (n = 30) while fifteen studies reported on alcohol use (n = 15). The median score for tobacco use (smoke and/or smokeless form) was 18.6% (IQR = 10.6%-23.8%) and 25.2% (IQR = 13.8%-38.1%) for alcohol consumption among the participants.

### Domains of T2DM self-care behaviours

Among all the self-care behaviours, physical activity (n = 61) was the most reported self-care behaviour followed by medication intake (n = 57), dietary habits (n = 56), self-monitoring of blood glucose (n = 42), and foot care (n = 30). Studies adopted a wide range of scales (n = 58) to assess the different domains of self-care behaviours. Many studies did not provide information about the tool used (n = 27) and some studies (n = 7) reported using author-developed tools. The Summary of Diabetes Self-Care Activities measure (SDSCA) [122] was used by 16 studies and the Diabetes Self-Management Questionnaire (DSMQ) [123] by seven studies to assess the different domains of self-care behaviours. Similarly, the Morisky Medication Adherence Scale (MMAS) scale was used by 23 studies in assessing the status of medication adherence among the study participants [124,125]. Four studies used the Global Physical Activity Questionnaire (GPAQ) [126] and the International Physical Activity Questionnaire (IPAQ) [127] was used by three studies to measure the physical activity level of the study participants. The findings on self-care behaviours were reported in the form of a percentage, mean and median.

#### Physical activity

Physical activity was assessed by 61 studies (**Table 2**). The Summary of Diabetes Self-Care Activities measure (n = 15), the Diabetes Self-Management Questionnaire (n = 7), the Global Physical Activity Questionnaire (n = 4), and the International Physical Activity Questionnaire (n = 3) were the most used tools in assessing the study participants’ physical activity. Two studies reported the mean number of days in a week participants were physically active, ranging from 4.08 to 4.23 days [20,45]. Additionally, four studies reported the mean score for physical activity ranging from 3.74 to 5.1 (scale range of 0-10, where 10 represents the optimal practice of self-care) [73,76,86,95].

**Table 2:** Physical activity

Authors	Year	Country	Sample size	Measure	Practice rates
Malathy et al.[36]	2011	India	207	Performing exercise regularly (Self-reported)	41%
Gopichandran et al. [37]	2012	India	200	Good exercise behaviour (at least 20 min a day exercise for 5 d in last week)	19.5%
Patel et al. [38]	2012	India	399	Following recommended Physical Activity	54%
Sasi et al. [39]	2013	India	546	Performing physical exercise for at least 30 min a day and 5 d a week	37%
Arulmozhi and Mahalakshmy [40]	2014	India	150	Physical exercise for at least 30 min for at least 4 d/week	22.7%
Santhanakrishnan, Lakshminarayanan and Kar [42]	2014	India	135	Practicing Physical Activity	37.0%
Saurabh et al. [43]	2014	India	103	Performing Physical Activity in addition to their routine work	45.6%,
Sajith et al. [44]	2014	India	105	Exercise adherence	32.3%,
Abraham et al. [45]	2015	India	60	Mean (SD) number of days in a week performing at least 30 min of physical activity or exercise	4.1 (±2.8)
Basu et al. [47]	2015	India	385	Specific exercise session averaging 30 min/d	3.6 (±2.3)
				a. <5 d (non-adherent) in the previous 7 d	52%
				b. ≥5 d (adherent) in the previous 7 d	48%
Rajasekharan et al. [48]	2015	India	290	Practicing Physical Activity of at least 30 min on all days of the week	43.4%
Das et al. [49]	2016	India	232	Exercise being done regularly	53.9%
Dinesh, Kulkarni and Gangadhar [52]	2016	India	400	Exercising at least 5 d a week for 20-30 min	20.5%
Debnath et al. [53]	2017	India	450	Performing good physical activity (regular walking)	38%
Sheeba, Ak and Biju [56]	2017	India	100	Performing regular exercise	46%
Srinath, Basavegowda and Tharuni [57]	2017	India	400	Participated in walking in the last week	27.7%
Britto et al. [58]	2018	India	25	Inactive	20%
				Moderately active	52%
				Highly active	28%
Pati et al. [59]	2018	India	321	Performing Physical Activity frequently	59%
Ravi, Kumar and Gopichandran [60]	2018	India	200	Median number of days in the past week participating in at least 30 min of physical activity	0 (IQR:0-7)
Jasmine and Iyer [62]	2019	India	77	Following regular physical exercise	15.6%
Aravind, Joy and Rakesh [64]	2019	India	68	Good physical activity	39.7%
Banerjee et al. [65]	2019	India	347	High level of physical activity	34.9%
				Moderate level of physical activity	31.1%
				Low level of physical activity	34%
Raj, Selvaraj and Thomas [66]	2019	India	205	Low Physical Activity	61.5%
				Moderate Physical Activity	19.5%
				High Physical Activity	19.0%
Sirari et al. [67]	2019	India	60	Performing at least 30 min of Physical Activity	61.3%
				Performing specific exercise session	48.4%
Bashir et al. [68]	2020	India	203	Mean (SD) score for daily exercising	2.6 (±0.9)
				Performing daily exercise as recommended	38.9%
Chandrika et al. [69]	2020	India	208	Performed physical activity for at least 30 min for minimum 5 d in the last week	30.3%
Chandrika et al. [69]	2020	India	208	Performed physical activity for at least 30 min for minimum 5 d in the last week	30.3%
Shrivastva et al. [73]	2020	India	166	Mean (SD) score for physical activity	4.9 (±2.8),
Karthik et al. [75]	2020	India	250	Performing satisfactory level of exercise	19.2%
Kumar et al. [76]	2021	India	105	Mean (SD) score for physical activity	5.1 (±1.6)
Rana et al. [77]	2021	India	200	Mean (SD) score adhering the exercise	1.2 (±1.3)
Verma et al. [18]	2021	India	416	Performing physical activity	72%
Burman et al. [78]	2021	India	367	Performing satisfactory level of exercise for at least 30 min in a week	76.5%
Durai et al. [79]	2021	India	390	Performing physical activity (at least 30 min for 3 or more days a week)	46%
Zuberi, Syed and Bhatti [83]	2011	Pakistan	286	Compliant with exercise	28.0%
Ahmed et al. [84]	2015	Pakistan	139	Following regular physical activity	8.6%
Javaid et al. [85]	2016	Pakistan	120	Low physical activity	67.0%
				Moderate physical activity	33.0%
Bukhsh et al. [86]	2017	Pakistan	130	Mean (SD) score for physical activity	4.0 (±3.1)
Bukhsh et al. [90]	2018	Pakistan	218	Median (IQR) score for physical activity	3.3 (1.11–6.67)
Siddique et al. [94]	2020	Pakistan	154	Performing exercise daily for 30 min	27.9%
Malik et al. [19]	2020	Pakistan	363	Exercising at least 20-30 min per day for at least five days a week	65.3%
Sayeed et al. [95]	2020	Pakistan	317	Mean (SD) score for physical activity	3.7 (±1.03)
Parajuli et al. [98]	2014	Nepal	385	Mean (SD) score for adherence to Physical Activity	67 (±23.9)
				a. Non-adherence	42.1%
				b. Poor adherence	36.6%
				c. Good adherence	21.0%
Sharma and Bhandari [99]	2014	Nepal	100	Exercise frequency	
				a. Daily	72.0%
				b. 2-3 d a week	18.0%
				c. 4-5 d a week	10.0%
				Exercise duration	
				a. 20 min	22.0%
				b. 30 min	30.0%
				c. 60 min	48.0%
Bhandari and Kim [20]	2016	Nepal	230	Mean (SD) number of days in a week performing exercise	4.2(±2.8)
Ghimire [100]	2017	Nepal	197	Non-compliant to exercise recommendation	46.0%
Shrestha et al. [101]	2017	Nepal	183	Performing physical exercise	67.7%
Kadariya and Aro [103]	2018	Nepal	270	Low level of physical activity	20.4%
				Medium level of physical activity	51.8%
				High level of physical activity	27.8%
Ghimire and Devi [102]	2018	Nepal	115	Performing good physical activity	56.5%
Sapkota et al. [104]	2018	Nepal	200	Performing exercise regularly	27%
Thapa [105]	2018	Nepal	141	Performing exercise regularly	56.7%
Pokhrel et al. [106]	2019	Nepal	480	High adherence to exercise	38.3%
Bhattarai et al. [107]	2019	Nepal	214	Not performing exercise regularly	63.6%
Shrestha et al. [109]	2021	Nepal	354	Performing physical activity	44%
Kandel et al. [110]	2022	Nepal	411	Recreational physical activity 7 d a week	48.2%
Saleh et al. [111]	2012	Bangladesh	160	Performing exercise	23.0%
Mumu et al. [112]	2014	Bangladesh	374	Non-adherence to exercise (<30 min a day):	25.0%
Saleh et al. [113]	2014	Bangladesh	500	Non-adherence to exercise (exercise <45 min/d)	33.2%
Chowdhury et al. [115]	2018	Bangladesh	11917	Performing regular exercise (more than 30 min/ at least 5 d per week)	69.0%
Bukht et al. [116]	2019	Bangladesh	977	Inactive/low (<150 min/week)	74.0%
				Moderate-to-vigorous (≥150minutes/week)	26.0%
Islam et al. [118]	2020	Bangladesh	265	Walk (30 min/d) for at least 5 d (last week)	70.9%
MahmudulHasan et al. [119]	2021	Bangladesh	379	Adherence to recommended Physical Activity (≥150 min in 7 d)	38.5%
Medagama and Galgomuwa [120]	2018	Sri Lanka	400	Physically inactive	21.5%
				Minimally active	33.8%
				Physically active	44.8%

SD – standard deviation, IQR – interquartile range

**Figure 2:**
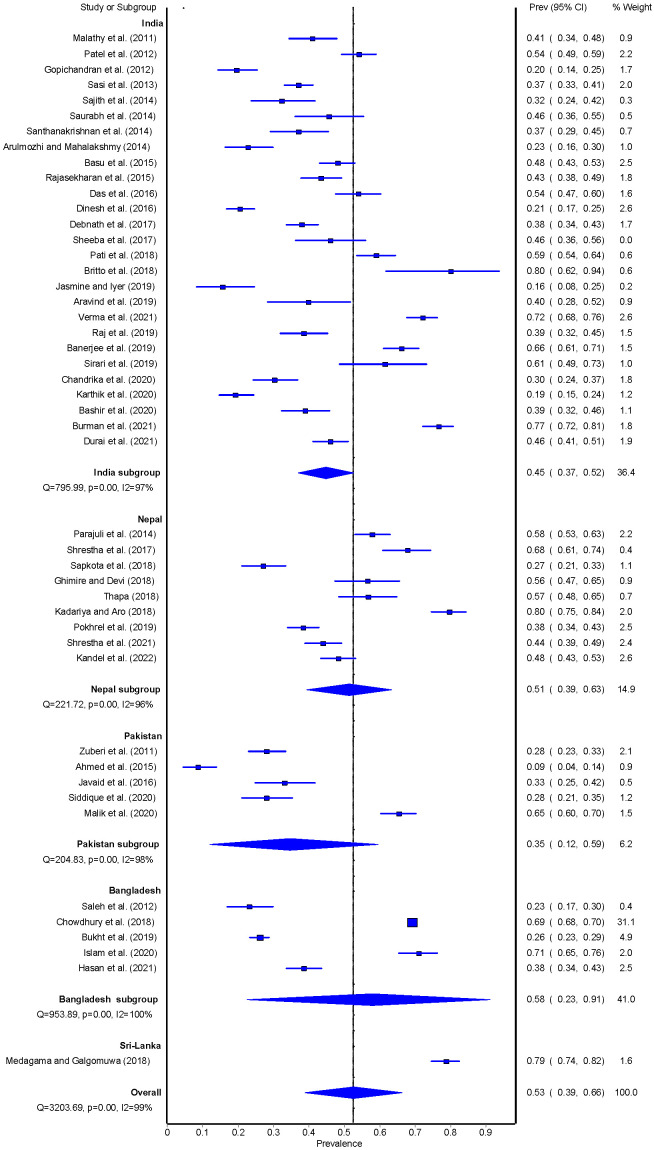
Pooled estimate of physical activity among people with T2DM.

The overall pooled prevalence of adherence to sufficient physical activity was 53% (95% CI = 39-66) and ranged from 9% to 80%. In terms of country-specific pooled prevalence, studies conducted in Sri Lanka (n = 1) reported an adherence of 79% (95% CI = 74-82), followed by Bangladesh (n = 5; 58%, 95% CI = 23-91), Nepal (n = 9; 51%, 95% CI = 39-63), India (n = 27; 45%, 95% CI = 37-52) and Pakistan (n = 5; 35%, 95% CI = 12-59) (**Figure 2**). Adherence to sufficient physical activity was 54% (95% CI = 38%-69%) for studies that either used a standardised tool to assess physical activity or studies that clearly defined what sufficient physical activity constitutes. Adherence to sufficient physical activity was 47% (95% CI = 34%-59%) for studies that did not clarify either the measure used to assess physical activity or studies that did not define what sufficient physical activity constitutes (Figure S1-S2 in the **Online Supplementary Document**).

#### Medication use

57 studies measured adherence to the medication use **(****Table 3****)**. The Morisky Medication Adherence Scale (n = 23) and the Summary of Diabetes Self-Care Activities measure (n = 10) were the most used tools in measuring adherence to medication use. A study from Nepal reported a mean of 6.77 number of days per week participants’ adhering to medication [20]. Non-adherence to Oral Hypoglycaemic Agents and Insulin was assessed by a study from Bangladesh [113], where 20% and 6.6% were non-adherence to Oral Hypoglycaemic Agents and Insulin respectively.

**Table 3:** Medication adherence

Authors	Year	Country	Sample size		Measures	Practice rates
Sultana et al. [35]	2010	India	218		Good adherence to medication	47.7%
Malathy et al.[36]	2011	India	207		Regularly taking the doses of diabetes medication	58.4%
Gopichandran et al. [37]	2012	India	200		Drug adherence	79.8%
Sasi et al. [39]	2013	India	546		Good adherence to medication	61%
					Poor adherence to medication	39%
Arulmozhi and Mahalakshmy [40]	2014	India	150		Low adherence	26%
					Moderate adherence	24.7%
					High adherence	49.3%
Khan et al. [41]	2014	India	184		Good adherence with the prescribed therapy	48.4%
Santhanakrishnan, Lakshminarayanan and Kar [42]	2014	India	135		Compliance to pharmacological treatment	76.3%
Sajith et al. [44]	2014	India	105		Low adherence	21.9%
					Moderate adherence	37.1%
					High adherence	40.9%
Basu et al. [47]	2015	India	385		Good medication adherence	74.5%
					Poor medication adherence	25.5%
Divya and Nadig [46]	2015	India	150		Non-adherence (low)	54.7%
					Adherence (Moderate-high)	45.3%
Rajasekharan et al. [48]	2015	India	290		Adherence to OHA's on all days of the week	60.5%
					Adherence to insulin injections on all days of the week	66.9%
Das et al. [49]	2016	India	232		Medicines taken regularly	90.5%
Karthikeyan, Madhusudhan and Selvamuthukumaran [50]	2016	India	345		Low adherence	95.6%
					Moderate adherence	4.3%
					High adherence	0
Pathania et al. [51]	2016	India	48		Low adherence	56.2%
					Moderate adherence	29.2%
					High adherence	14.6%
Dinesh, Kulkarni and Gangadhar [52]	2016	India	400		Taking drugs every day and regularly	48%
Debnath et al. [53]	2017	India	450		Good medication adherence	38%
					Poor medication adherence	62%
Kumar et al. [54]	2017	India	124		Low adherence	43.5%
					Moderate adherence	29%
					High adherence	27.4%
Samu, Amirthalingam and Mohammed [55]	2017	India	86		Low medication adherence	4.3 (±2.3)
Sheeba, Ak and Biju [56]	2017	India	100		Taking regular medication	88%
Srinath, Basavegowda and Tharuni [57]	2017	India	400		Good compliance for medication	92.5%
Venkatesan, Dongre and Ganapathy [61]	2018	India	328		Low adherent for medication	45.4%
Acharya et al. [63]	2019	India	200		Low adherence	33%
					Moderate adherence	34.5%
					High adherence	32.5%
Jasmine and Iyer [62]	2019	India	77		Good compliance to treatment	64.9%
					Poor compliance to treatment	35.1%
Chandrika et al. [69]	2020	India	208		Good drug adherence	56.3%
Kowsalya et al. [70]	2020	India	60		Low adherence	2%
					Moderate adherence	20%
					High adherence	78%
Palathingal et al. [71]	2020	India	200		Low adherence	71.5%
					Moderate adherence	24%
					High adherence	4.5%
Achappa [74]	2020	India	70		Good adherence to medication	80%
					Poor adherence to medication	20%
Karthik et al. [75]	2020	India	250		Low adherence	29.6%
					High adherence	70.4%
Rana et al. [77]	2021	India	200		Mean (SD) score adhering the medication	0.3 (±0.7)
Burman et al. [78]	2021	India	367		Taking medication daily	93%
Durai et al. [79]	2021	India	390		Adherence to medication	57.2%
Mishra et al. [80]	2021	India	277		Good adherence	44%
					Poor adherence	56%
Singh et al. [81]	2021	India		350	Low adherence	26%
					Moderate adherence	42%
					High adherence	32%
Aravindakshan et al. [82]	2021	India	218		Low adherence	10.5%
					Moderate adherence	29.4%
					High adherence	60.1%
Zuberi, Syed and Bhatti [83]	2011	Pakistan	286		Taking dose on time	84%
					Taking recommended dose of medication	83%
Ahmed et al. [84]	2015	Pakistan	139		Taking medication on time	7.9%
Iqbal et al. [87]	2017	Pakistan	300		Low adherence	7.3%
					Moderate adherence	37%
					High adherence	55.6%
Nazirl et al. [88]	2017	Pakistan	392		Low adherence	71.9%
					Moderate adherence	24.7%
					High adherence	3.32%
Rana et al. [89]	2017	Pakistan	145		Low adherence	19.3%,
					Moderate adherence	43.4%
					High adherence	37.2%
Hussain, Said and Khan [93]	2020	Pakistan	524		Mean (SD) score adhering the medication	3.1 (±0.5)
Siddique et al. [94]	2020	Pakistan	154		Taking medication daily	74%
Malik et al. [19]	2020	Pakistan	363		Daily medication use	66.4%
Ishaq et al. [96]	2021	Pakistan	300		Low adherence	7.3%
					Moderate adherence	37%
					High adherence	55.6%
Shrestha et al. [97]	2013	Nepal	100		Non-adherence to medication	38%
Bhandari and Kim [20]	2016	Nepal	230		Mean (SD) number of days in a week adhering the medication	6.8(±1.1)
Shrestha et al. [101]	2017	Nepal	183		Adherence to medication	77%
Sapkota et al. [104]	2018	Nepal	200		Forgot to take diabetes tablet/insulin in the last year	
					a. <5 times	76%
					b. ≥5 times	24%
Thapa [105]	2018	Nepal	141		Adherence to OHA on 7 d of a week	86.5%
					Adherence to insulin on 7 d of the week	78%
Pokhrel et al. [106]	2019	Nepal	480		Low adherence	36.6%
					High adherence	63.4%
Bhattarai et al. [107]	2019	Nepal	214		Adherence to medication	44.9%
					Non-adherence to medication	55.1%
Sharma et al. [108]	2021	Nepal	296		Adherence to medication	86.8%
Shrestha et al. [109]	2021	Nepal	354		Adherence to medication	92%
Kandel et al. [110]	2022	Nepal	411		Adherence to OHA	98.2%
					Adherence to insulin	100%
Saleh et al. [113]	2014	Bangladesh	500		Non-adherence to OHA	20%
					Non-adherence to insulin	6.6%
Ahmed et al. [114]	2017	Bangladesh	122		Taking medication regularly as prescribed	43%
					Taking medication irregularly	57%
Mannan et al. [21]	2021	Bangladesh	2061		Low adherence	46.3%
					Medium- to-high adherence	53.7%
Rathish et al. [121]	2019	Sri-Lanka	200		Low adherence	7%
					Moderate adherence	70%
					High adherence	23%

SD – standard deviation, OHA – oral hypoglycaemic agent, d – day

**Figure 3:**
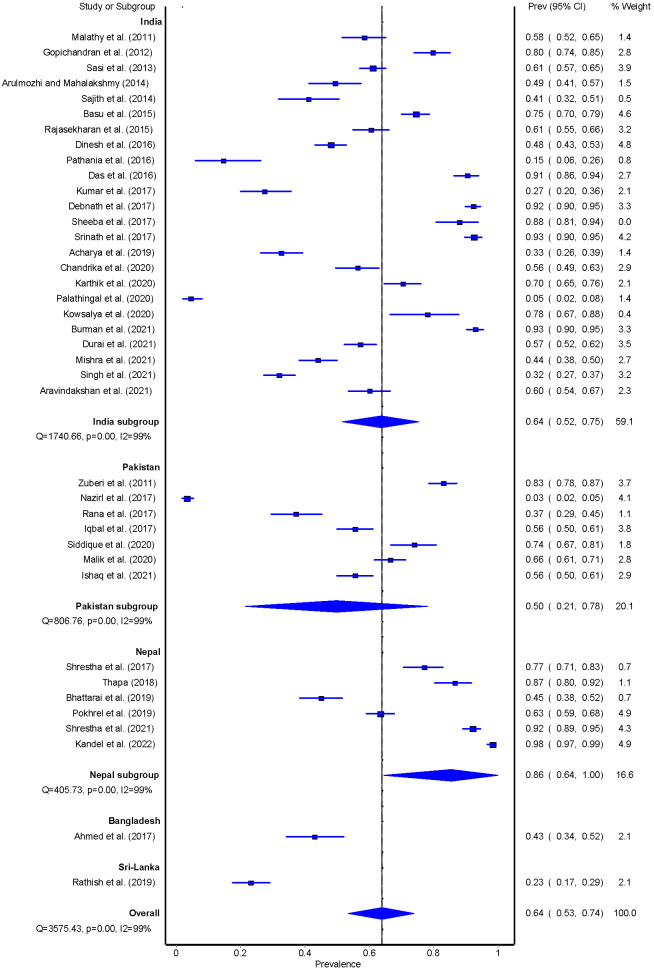
Pooled estimate of medication intake among people with T2DM.

The pooled prevalence of adherence to medication use was 64% (95% CI = 53-74) and ranged between 3% and 98%. Studies conducted in Nepal reported a higher prevalence of adherence to medication use (n = 6; 86%, 95% CI = 64-100), followed by India (n = 19; 64%, 95% CI = 52-75), Pakistan (n = 6; 50%, 95% CI = 21-78), Bangladesh (n = 1; 43%, 95% CI = 34-52) and Sri-Lanka (n = 1; 23%, 95% CI = 17-29) **(****Figure 3****)**.

#### Dietary habits

56 studies explored the study participants’ dietary intake (**Table 4**) using a range of dietary measurement tools. Summary of the Diabetes Self-Care Activities measure (n = 15) and the Diabetes Self-Management Questionnaire (n = 7) were the most used self-care tools in assessing the dietary practice of the study participants. Some studies reported the mean number of days in a week participant’s adhering to a healthy diet ranging from 4.32 to 5.42 days [20,45,47]. In addition, the reported mean score for dietary control (limiting sweets and carbohydrate-rich foods, consuming recommended diet) varied from 3.9 to 6.6 (scale range of 0-10, where 10 represents the optimal practice of self-care) [73,76,86,95]. Non-adherence to healthy dietary habits was reported by four studies whose values range from 41% to 88% [100,107,112,113].

**Table 4:** Dietary habits

Authors	Year	Country	Sample size	Measure	Practice rates
Shah, Kamdar and Shah [34]	2009	India	238	Including fruits in diet regularly	54.2%
				Taking green leafy vegetables in diet	31.9%
Malathy et al.[36]	2011	India	207	Following a controlled and planned diet (self-reported)	50%
Gopichandran et al. [37]	2012	India	200	Having good dietary behaviour	29%
Patel et al. [38]	2012	India	399	Following the recommended diabetic diet	73%
Sasi et al. [39]	2013	India	546	Following the diabetic meal plans	41%
Arulmozhi and Mahalakshmy [40]	2014	India	150	Consumed recommended diet for at least 4 d/week	67.3%
Santhanakrishnan, Lakshminarayanan and Kar [42]	2014	India	135	Reduced the quantity of food intake	77%
				Increased frequency of food intake	50.3%
Saurabh et al. [43]	2014	India	103	Following the diet-control	58.3%
Sajith et al. [44]	2014	India	105	Dietary adherence	3.8%
Abraham et al. [45]	2015	India	60	Mean number of days in a week following general diet*	5.3
				Mean number of days in a week following specific diet†	5.4
Basu et al. [47]	2015	India	385	Mean (SD) number of days in a week following a healthy eating plan	4.8 (±1.4)
Rajasekharan et al. [48]	2015	India	290	Following healthy eating plan on all days of the week	45.9%
				Incorporating fruits/vegetables in the diets on all days of the week	26.2%
Das et al. [49]	2016	India	232	Following the planned and the controlled diet	76.3%
Dinesh, Kulkarni and Gangadhar [52]	2016	India	400	Having a good dietary behaviour	24%
Sheeba, Ak and Biju [56]	2017	India	100	Following the proper diet	72%
Srinath, Basavegowda and Tharuni [57]	2017	India	400	Compliant to diabetic diet as advised by the doctor	72.0%
				Had vegetables on all seven days in the last week	96.2%
				Consuming fruits on all seven days in the last week	5.5%
Pati et al. [59]	2018	India	321	Following the strict diabetic diet control	45%
Ravi, Kumar and Gopichandran [60]	2018	India	200	Median (IQR) number of days following healthy eating plan in the past week	6 (2-6)
				Median (IQR) number of days in the past week taking five or more servings of fruits/vegetables	0 (0)
Aravind, Joy and Rakesh [64]	2019	India	68	Following good diet	45.6%
Jasmine and Iyer [62]	2019	India	77	Good diabetic diet practice	44.9%
Sirari et al. [67]	2019	India	60	Compliant for not eating high-fat foods	93.5%
				Compliant with prescribed eating plan	51.6%
				Compliant with eating 5 or more servings of fruits and vegetables	59.7%
Bashir et al. [68]	2020	India	203	Mean (SD) score of consumption of healthiest diet	1.0 (±0.2)
				Mean (SD) score of consumption of least healthy diet	2.6 (±0.7)
Chandrika et al. [69]	2020	India	208	Good dietary behaviour	29.8%
Patnaik et al. [72]	2020	India	100	Follow instructions provided to avoid certain foods	77%
				Follow the recommended amount of diet	67%
				Taking sweets	38%
Shrivastva et al. [73]	2020	India	166	Mean (SD) score for dietary control:	6.6 (±1.9)
Karthik et al. [75]	2020	India	250	Following satisfactory level of diet:	35.2%
Kumar et al. [76]	2021	India	105	Mean (SD) score of dietary control‡	5.7 (±1.5)
Rana et al. [77]	2021	India	200	Mean (SD) score adhering the diet:	1.1 (±0.8)
Burman et al. [78]	2021	India	367	Consumption of satisfactory level of fruits and vegetables in last 7 d:	61.5%
Durai et al. [79]	2021	India	390	Adherent to dietary modifications:	25.4%
Zuberi, Syed and Bhatti [83]	2011	Pakistan	286	Complying with the dietary restrictions:	61.2%
Ahmed et al. [84]	2015	Pakistan	139	Following a proper diet plan:	4.3%
Javaid et al. [85]	2016	Pakistan	120	Good dietary practice:	71.7%
Bukhsh et al. [86]	2017	Pakistan	130	Mean (SD) value of dietary control:	4.8 (±2.8)
Bukhsh et al. [90]	2018	Pakistan	218	Median (IQR) score for dietary control:	4.17 (2.5– 6.9)
Farooq et al. [91]	2018	Pakistan	180	Strictly following a recommended dietary plan:	36.1%
				Changing diet following diabetes diagnosis:	82.2%
				Taking three meals a day:	55.6%
				Eating same meal as their family:	79.4%
Siddique et al. [94]	2020	Pakistan	154	Following the dietary plan daily:	50%
Malik et al. [19]	2020	Pakistan	363	Following well-balanced and planned diet:	68.9%
Sayeed et al. [95]	2020	Pakistan	317	Mean (SD) score for dietary control:	3.87 (±1.04)
Parajuli et al. [98]	2014	Nepal	385	Dietary advice:	30.0 (±16.3)
				a. Non-adherence	87.5%
				b. Poor adherence	12.5%
				c. Good adherence	0%
Sharma and Bhandari [99]	2014	Nepal	100	Food intake per day:	
				a. Two times	20%
				b. Three times	42%
				c. Four times	38%
Bhandari and Kim [20]	2016	Nepal	230	Mean (SD) number of days in a week adhering the diet:	4.3(±1.4)
Ghimire [100]	2017	Nepal	197	Non-compliant with the dietary recommendation	41%
Shrestha et al. [101]	2017	Nepal	183	Dietary habits:	
				a. Vegetarian	12%
				b. non-vegetarian	88%
Ghimire and Devi [102]	2018	Nepal	115	Having good dietary management	47%
Thapa [105]	2018	Nepal	141	Following recommended dietary plans	95.7%
				Eating fruits and vegetables for at least 5 d/week	73.8%,
				Consuming high fat food	39%
Pokhrel et al. [106]	2019	Nepal	480	Adhering the recommended meal plan:	64.6%
Bhattarai et al. [107]	2019	Nepal	214	Not following the diabetic diet:	85.7%
Shrestha et al. [109]	2021	Nepal	354	Dietary adherence:	38%
Kandel et al. [110]	2022	Nepal	411	Ate ≥5 small meals every day in last 7 d	15.3%
				Ate >2 bowls of vegetables every day in last 7 d	78.3%
				Ate >1 bowl of fruits every day in last 7 d	45.3%
				Ate fatty food or red meat at most once in last 7 d	55.5%
				Refused offered sweets within the past 1 mo	70%
Saleh et al. [111]	2012	Bangladesh	160	Following dietary control:	18%
Mumu et al. [112]	2014	Bangladesh	374	Non-adherence to recommended diet plan:	88%
Saleh et al. [113]	2014	Bangladesh	500	Non-adherence to diet:	44.8%
Chowdhury et al. [115]	2018	Bangladesh	11 917	Taking food timely:	69%
				Have habit of extra salt intake:	69%
Majid et al. [117]	2019	Bangladesh	420	**A. Carbohydrate intake:**	**259.2 (±57.2)**
				a. low	5.7%
				b. ideal	36.2%
				c. high	58.1%
				**B. Protein intake:**	**87.2 (±19.1)**
				a. low	14.3%
				b. ideal	55.2%
				c. high	30.5%
				**C. Fat intake:**	**65.1 (±12.2)**
				a. low	1.9%
				b. ideal	42.9%
				c. high	55.2%
MahmudulHasan et al. [119]	2021	Bangladesh	379	Adherence to recommended diet	24.3%
Mannan et al. [21]	2021	Bangladesh	2061	Consumption of fruit and vegetables:	
				a. ≥3 times/d	4.9%
				b. <3 times/d	95.1%

SD – standard deviation, IQR – interquartile range, d – days

*General diet: Consumption of generally helpful or prescribed diet.

†Specific diet: Consumption of five or more servings of “fruits and vegetables” and avoiding fat foods.

‡Dietary control: Limiting sweets and carbohydrate rich foods, consuming recommended diet.

**Figure 4:**
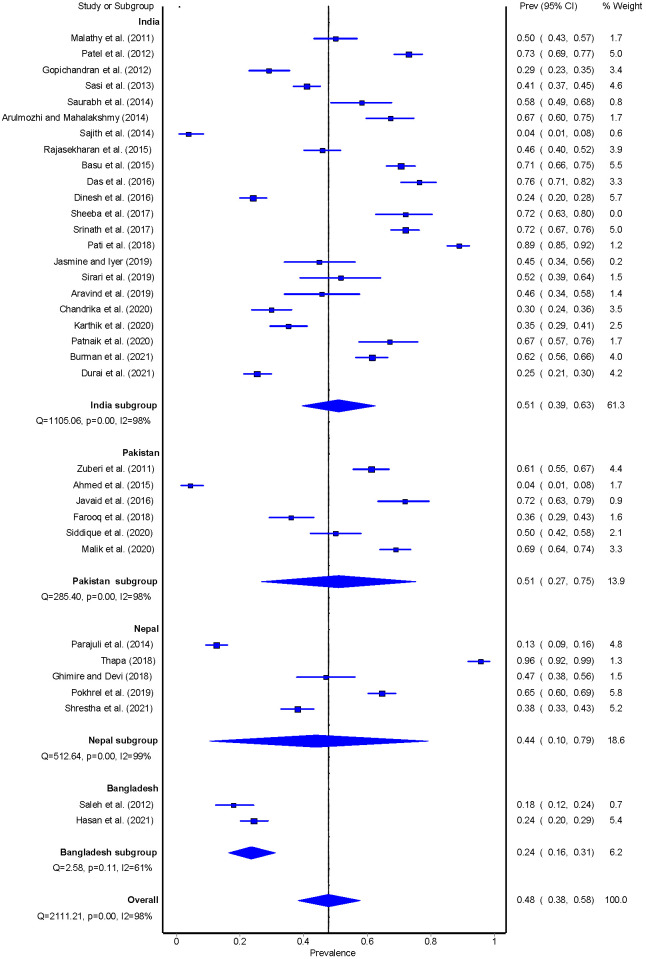
Pooled estimate of Dietary habit among people with T2DM.

The prevalence of adherence to a healthy diet varied widely across studies, from 0% to 95.7%. The overall pooled prevalence of adherence to a healthy diet was 48% (95% CI = 38-58). In terms of country-specific analysis, the studies conducted in India (n = 22) had an adherence to a healthy diet of 51% (95% CI = 39-63), followed by Pakistan (n = 6; 51%, 95%CI: 27-75), Nepal (n = 4; 44%, 95%CI: 10-79) and Bangladesh (n = 2; 24%, 95%CI: 16-31) (**Figure 4****)**. Adherence to a healthy diet was 40% (95%CI = 29%-53%) for studies that either used a standardised tool to assess diet or studies that clearly defined what a healthy diet constitutes. Adherence to a healthy diet was 57% (95%CI = 42%-72%) for studies that did not clarify either the measure used to assess diet or studies that did not define what a healthy diet constitutes (Figure S3-S4 in the **Online Supplementary Document**).

#### Blood glucose monitoring

42 studies investigated blood glucose monitoring (**Table 5**). The Summary of Diabetes Self-Care Activities measure (n = 10) and the Diabetes Self-Management Questionnaire (n = 7) were the most used tools in assessing the blood glucose monitoring among the study participants. The mean number of days in a week practicing adequate self-monitoring of blood glucose was reported by two studies and ranged from 0.61 to 1.33 days [20,45]. Similarly, the reported mean scores for glucose management ranged from 3.92 to 6.82 (scale ranging from 0 to 10, where 10 represents the highest practice of self-care behaviour) [73,76,86,95]. However, non-adherence to blood glucose monitoring was reported by a study from Bangladesh [112,113] which ranged from 32% to 37%, while 46.61% of participants did not monitor the glucose level regularly in Nepal [107].

**Table 5:** Blood glucose monitoring

Authors	Year	Country	Sample size	Measure	Practice rates
Shah, Kamdar and Shah [34]	2009	India	238	Checking blood glucose monthly	70.2%
Gopichandran et al. [37]	2012	India	200	Regular monitoring of blood glucoses (at least once in the previous 3 mo)	70%
Patel et al. [38]	2012	India	399	Self-monitoring blood glucose	37%
Saurabh et al. [43]	2014	India	103	Checking blood glucose at least once in 3 mo	75.7%
Abraham et al. [45]	2015	India	60	Mean (SD) number of days in a week testing the blood glucose*	1.3
Rajasekharan et al. [48]	2015	India	290	Blood glucose testing at least for once in past 3 mo	76.6%
Das et al. [49]	2016	India	232	Last checked blood glucose as advised	64.2%
Dinesh, Kulkarni and Gangadhar [52]	2016	India	400	Checking of blood glucoses at least once in 3 mo	65.2%
				Checking of blood glucoses as advised by doctor	72.7%
Debnath et al. [53]	2017	India	450	Blood glucose check-up	
				Good	48.7%
				Average	39.1%
				Poor	12.2%
Kumar et al. [54]	2017	India	124	Blood glucose monitoring:	
				a. Regular (once in a month)	75.8%
				b. Occasional	24.2%
Sheeba, Ak and Biju [56]	2017	India	100	Regularly monitoring blood glucose level	63%
Srinath, Basavegowda and Tharuni [57]	2017	India	400	Blood glucose check as advised by doctor	18.2%
Ravi, Kumar and Gopichandran [60]	2018	India	200	Median (IQR) score for blood glucose testing at least once in past 3 mo	1 (0-1)
Aravind, Joy and Rakesh [64]	2019	India	68	Good glucose management	52.9%
Jasmine and Iyer [62]	2019	India	77	Regular blood glucose check-up at Primary Health Center	
				a. good practice	88.3%
				b. poor practice	11.7%
Sirari et al. [67]	2019	India	60	Blood glucose monitoring at least once in every 3 mo	91.9%
Chandrika et al. [69]	2020	India	208	Blood glucose monitoring at least once within the previous 3 mo	44.2%
Palathingal et al. [71]	2020	India	200	Blood glucose monitoring:	
				a. once in a month	46%
				b. once in three months	46%
				c. once in six months	7%
				d. once a year	1%
Shrivastva et al. [73]	2020	India	166	Mean (SD) score for glucose management	6.8 (±1.7)
Karthik et al. [75]	2020	India	250	Regularly monitoring/checking-up the blood glucose	75.2%
Kumar et al. [76]	2021	India	105	Mean (SD) score for glucose management	5.7(±1.1)
Rana et al. [77]	2021	India	200	Mean (SD) score adhering the self-monitoring of blood glucose	0.3 (±0.8)
Burman et al. [78]	2021	India	367	Checking blood glucose level in the past 3 mo	95%
Durai et al. [79]	2021	India	390	Blood glucose testing once in 3 mo	90%
Ahmed et al. [84]	2015	Pakistan	139	Regularly checking blood glucose level at home	8.6%
Bukhsh et al. [86]	2017	Pakistan	130	Mean (SD) score for glucose management	5.3 (±2.9)
Bukhsh et al. [90]	2018	Pakistan	218	Median (IQR) score for glucose management	4.7 (3.3–7.3)
Siddique et al. [94]	2020	Pakistan	154	Monitoring glucose twice a week	54.5%
Malik et al. [19]	2020	Pakistan	363	Checking blood glucose at home as per health practitioners	69.7%
				Checking HbA1c levels every three months	28.4%
				Checking random blood glucose level at least once every three months	65.8%
Sayeed et al. [95]	2020	Pakistan	317	Mean (SD) score for glucose management	3.9 (±0.6)
Sharma and Bhandari [99]	2014	Nepal	100	Blood glucose test:	
				a. once a week	2%
				b. once a month	82%
				c. half yearly	16%
Bhandari and Kim [20]	2016	Nepal	230	Mean (SD) number of days in a week monitoring the blood glucose	0.6(±0.9)
Ghimire and Devi [102]	2018	Nepal	115	Good glucose management practice	68.2%
Sapkota et al. [104]	2018	Nepal	200	Checking blood glucose	
				a. once within a day to 1 mo	19%
				b. once within a month to 1 y	81%
Thapa [105]	2018	Nepal	141	Monitoring blood glucose in every 3 mo	69.5%
Pokhrel et al. [106]	2019	Nepal	480	Blood glucose monitoring:	
				a. weekly	2.1%
				b. monthly	48.3%
				c. triannual	31.2%
				d. biannual	14.6%
				e. yearly	3.8%
Bhattarai et al. [107]	2019	Nepal	214	Not monitoring the blood glucose level regularly	46.6%
Shrestha et al. [109]	2021	Nepal	354	Optimal blood glucose testing	77%
Kandel et al. [110]	2022	Nepal	411	Blood glucose testing at least 3 times in the last 7 d	14.4%
Mumu et al. [112]	2014	Bangladesh	374	Non-adherence to blood glucose monitoring (missing the scheduled blood testing)	32%
Saleh et al. [113]	2014	Bangladesh	500	Non-adherence to blood glucose monitoring	37%
Ahmed et al. [114]	2017	Bangladesh	122	Blood glucose monitoring:	
				a. Daily	8%
				b. Weekly	15%
				c. Monthly	37%
				d. Never	40%
Chowdhury et al. [115]	2018	Bangladesh	11 917	Blood glucose monitoring:	
				a. Daily	6%
				b. Weekly	1%
				c. Monthly	65%
				d. Never	28%
Islam et al. [118]	2020	Bangladesh	265	Self-monitoring of blood glucose at home:	
				a. Weekly	12.4%
				b. Monthly	30.6%
				c. Every 2-3 mo or later	57%

IQR – interquartile range, SD – standard deviation, HbA1c – glycated haemoglobin, d – days, mo – months

*Testing blood glucose: Testing of blood glucose and as recommended by health care provider

**Figure 5:**
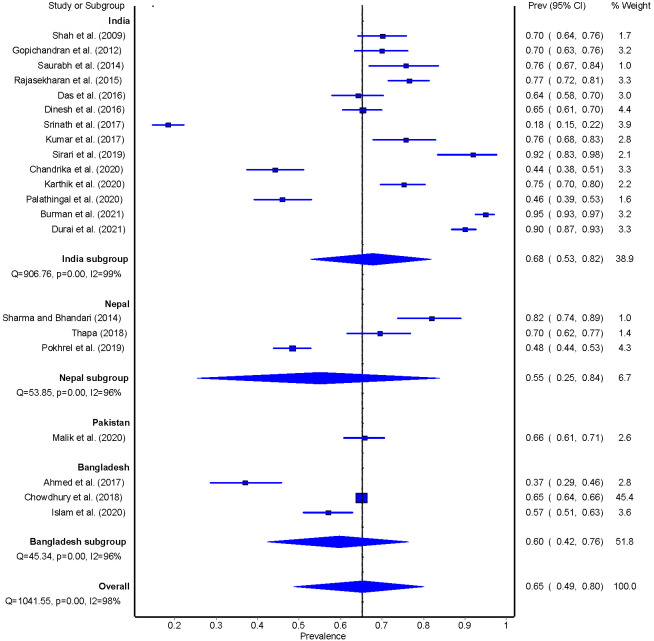
Pooled estimate of blood glucose monitoring among people with T2DM.

The overall pooled prevalence of blood glucose monitoring was 65% (95%CI: 49-80), ranging between 18% to 95%. A higher prevalence of adequate blood glucose monitoring among people with T2DM was found in India (n = 14; 68%, 95% CI = 53-82), followed by Pakistan (n = 1; 66%, 95% CI = 61-71), Bangladesh (n = 3; 60%, 95% CI = 42-76), and Nepal (n = 3; 55%, 95% CI: 25-84) (**Figure 5**). The sub-group analysis for blood glucose monitoring was assessed based on monitoring blood glucose levels at least once a month and/or at least once in three months.

In the sub-group analysis, the pooled prevalence of monthly blood glucose monitoring was 63% (95% CI = 48-77), while it was 67% (95% CI = 53-79) for the at least once in three months interval (Figure S5-S6 in the **Online Supplementary Document**).

#### Foot care

Thirty studies investigated the practice of foot care (**Table 6**). The Summary of Diabetes Self-Care Activities measure tool (n = 10) was most used in assessing the study participants’ foot care. The mean number of days in a week participants practicing foot care ranged between 0.55 and 2.16 days [20,45]. A study from India among 200 people with T2DM reported that the median number of days in the past week inspecting shoes or footwear was “0” [60]. Studies from Bangladesh reported on non-adherence to foot care ranging from 43.2 to 70% [112,113].

**Table 6:** Foot care

Authors	Year	Country	Sample size	Measures	Practice rates
Shah, Kamdar and Shah [34]	2009	India	238	Checking the feet regularly	56%
Sasi et al. [39]	2013	India	546	Adequate foot care	31%
				Inadequate foot care	69%
Arulmozhi and Mahalakshmy [40]	2014	India	150	Inspected foot at least 4 d/week:	22.7%
				Foot care at least 4 d/week (drying between toes after wash)	24%
Santhanakrishnan, Lakshminarayanan and Kar [42]	2014	India	135	Practicing foot care	54%
Saurabh et al. [43]	2014	India	103	Daily inspection of feet or their footwear	47.6%
				Daily washing and drying of feet	80.6%
				Poor practice of foot care	44.7%
				Satisfactory practice of foot care	35.9%
				Good practice of foot care	19.4%
Abraham et al. [45]	2015	India	60	Mean (SD) number of days in a week practicing foot care*	0.6
Rajasekharan et al. [48]	2015	India	290	Washing feet on all days of the week	64.8%
				Drying between the toes on all days of week	70.7%
				Examining feet on all days of the week	28.3%
				Inspecting the inner surface of shoes on all days of the week	13.4%
Das et al. [49]	2016	India	232	Regularly practicing the foot care	55.6%
Dinesh, Kulkarni and Gangadhar [52]	2016	India	400	Checking the feet daily	0.5%
				Inspecting inside of shoes/footwear daily	0.5%
Debnath et al. [53]	2017	India	450	Practicing good foot care	6.2%
Sheeba, Ak and Biju [56]	2017	India	100	Performing proper foot care	79%
Srinath, Basavegowda and Tharuni [57]	2017	India	400	Checking feet daily (last week)	24.2%
Ravi, Kumar and Gopichandran [60]	2018	India	200	Median number of days in the past week checking feet	0 (IQR = 0)
				Median number of days in the past week inspecting inside of shoes	0 (IQR = 0)
Jasmine and Iyer [62]	2019	India	77	Good practice of inspecting feet	13%
				Good practice of using footwear	51.9%
Sirari et al. [67]	2019	India	60	Inspecting shoes from inside	66.1%
				Performing foot care checking feet	67.7%
Karthik et al. [75]	2020	India	250	Practicing satisfactory foot care	17.6%
				Practicing unsatisfactory foot care	82.4%
Verma et al. [18]	2021	India	416	Poor practice of foot care	20.6%
				Satisfactory practice of foot care	32.7%
				Good practice of foot care	46.7%
Burman et al. [78]	2021	India	367	Taking care of foot regularly	54.5%
Durai et al. [79]	2021	India	390	Inspecting the foots regularly	26.7%
Zuberi, Syed and Bhatti [83]	2011	Pakistan	286	Compliant with foot care	82%
Ahmed et al. [84]	2015	Pakistan	139	Proper cutting of nails	5.8%
Zafar et al. [92]	2018	Pakistan	220	Poor practice of foot care	24.1%
				Average practice of foot care	59.1%
				Good practice of foot care	16.8%
Malik et al. [19]	2020	Pakistan	363	Checking the feet daily	58.4%
Bhandari and Kim [20]	2016	Nepal	230	Mean (SD) number of days in a week practicing foot care	2.2(±2.4)
Thapa [105]	2018	Nepal	141	Washing feet daily	100%
				Habit of inspecting feet	92.2%
				Trim nails regularly	100%
				Drying the toes on all day of the week	78%
Shrestha et al. [109]	2021	Nepal	354	Optimum foot care	42%
Kandel et al. [110]	2022	Nepal	411	Checked feet every day in the last 7 d:	51.1%
Mumu et al. [112]	2014	Bangladesh	374	Non-adherence to foot care (not following the recommended foot care)	70%
Saleh et al. [113]	2014	Bangladesh	500	Non-adherence to foot care	43.2%
Islam et al. [118]	2020	Bangladesh	265	Practicing the foot care (last week)	37.4%

SD – standard deviation

*Foot care: Checking feet and inside of shoe, and washing, and drying feet.

**Figure 6:**
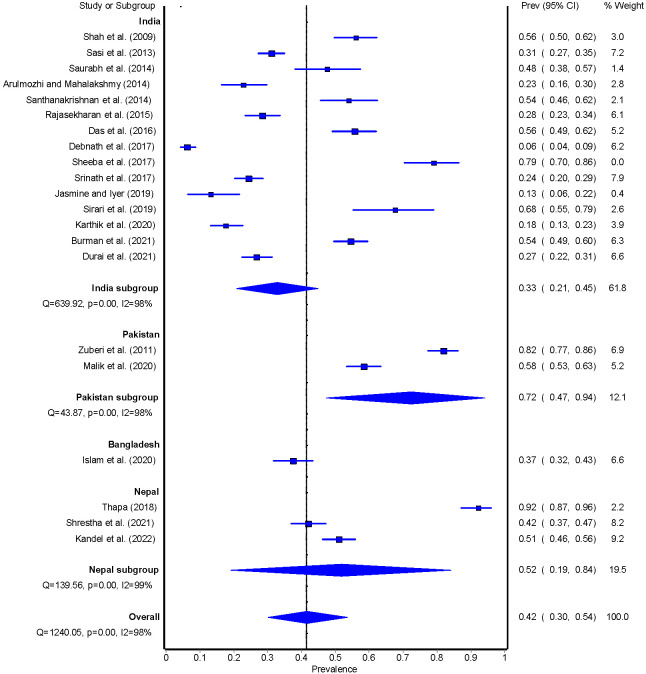
Pooled estimate of foot care among people with T2DM.

The overall pooled prevalence of adherence to foot care was 42% (95% CI = 30-54) and ranged between 6% and 92%. Studies conducted in Pakistan reported the highest adherence to foot care (n = 2; 72%, 95%CI = 47-94) followed by Nepal (n = 3; 52%, 95% CI = 19-84), Bangladesh (n = 1; 37%, 95% CI = 32-43) and India (n = 15; 33%, 95% CI = 21-45) (**Figure 6**). Sufficient adherence to foot care was 37% (95% CI = 18-57) for studies that either used a standardised tool to assess foot care or studies that clearly defined what adequate foot care constitutes. Adherence to sufficient foot care was 29% (95% CI = 8-57) for studies that did not clarify either the measure used to assess foot care or studies that did not define what sufficient foot care constitutes (Figures S7-S8 in the **Online Supplementary Document**).

## DISCUSSION

Adherence to self-care behaviour prevents T2DM-associated morbidities and mortalities [14,15]. The systematic reviews that included studies from Ethiopia [22,23], Sub-Saharan Africa [24], and LMICs [25] reported the poor practice of self-care behaviours among the people with T2DM and stressed the need for developing and implementing interventions to improve self-care behaviour. South Asians are at higher risk of developing NCDs, including type 2 diabetes [5], and the health care resources in this region are limited [128,129]. While previous systematic reviews on self-care behaviours have been conducted in various regions [11,22-25], no reviews have focused on studies conducted in South Asia. To the best of our knowledge, this is the first systematic review and meta-analysis to systematically assess and report the evidence on self-care behaviours among people with T2DM in South Asia countries. We also conducted a meta-analysis to calculate the pooled prevalence of different domains of self-care behaviour reported in 70 studies. The prevalence of T2DM self-care behaviour was highest for blood glucose monitoring, followed by medication adherence, physical activity, diet, and foot care.

Physical activity was the most frequently measured self-care behaviour among included studies. The pooled prevalence of adherence to physical activity was 53%. This shows insufficient participation in physical activity among South Asians with T2DM. Similar findings were observed in reviews from Ethiopia conducted by Dagnew et al. [23] (pooled prevalence = 48.29%) and Katema et al. [22] (pooled prevalence = 49%). In contrast, lower adherence to physical activity was reported in Canadian studies (21%) by Thiel et al. [130] and LMICs (41.2%) by Morge et al. [25]. This discrepancy might be because of the higher presence of manual labour in low-income countries compared to sedentary occupation and personal motorized transportation in high-income countries [131]. However, studies identified that time constraint, unwillingness, poor awareness level, comorbid conditions, social issues, lack of infrastructure and insufficient emphasis by physicians were the barriers to physical activity among South Asians with T2DM [59,100,120,132]. Engaging in sufficient physical activity reduces the risk of T2DM and plays a significant role in reducing the glycaemic level among people with T2DM [133,134]. This illustrates that physical activity is a key self-care behaviour for diabetes management and needs to be improved in South Asians with T2DM by designing culturally acceptable and person-centred interventions to facilitate and encourage people to adopt healthy behaviours.

The pooled prevalence of adherence to medication use in this review was 64%. This finding is consistent with the review from Sub-Saharan Africa conducted by Stephani et al. [24] that reported a mean adherence to medication use of 64% (range = 39%-88%). However, a higher prevalence of adherence to medication use (71%, range = 59%-83%) is reported by Morge et al. [25]. The lower adherence to medication use in South Asia might be the result of a poor understanding of the role of medication use in controlling blood glucose levels and the preference for traditional home remedies [135]. The practice of fasting, a cultural practice observed by people from various faiths, may impede compliance with medication intake [136,137]. Unaffordability, lack of information about prescribed medicines and their importance, ignorance and unwillingness, forgetfulness, poor drug supply from health facilities and poor doctor-patient relationship are significant factors for the lower adherence to medication use among the people with T2DM in South Asian countries [19,46,61,104,107]. Although adherence to medication use has a positive impact in controlling the glycaemic level [138], almost half of the South Asians did not adhere to their prescribed medication use and are at risk of developing acute and long-term complications, consequently leading to an increased hospitalization rate and higher medical costs [138-140]. Interventions aiming to raise awareness of the role of regular use of medication in controlling blood glucose levels are to be designed and implemented to adhere to the treatment regimen, and primary health care facilities need to be equipped with proper infrastructure required for screening of diabetes, provision of regular drug supplies and counselling services.

Less than half (48%) of the study participants adhered to healthy dietary behaviour. This finding is consistent with the reviews from Ethiopia conducted by Dagnew et al. [23] and Katema et al. [22], both reporting a pooled estimate of 50% for good dietary practice. A study by Stephani et al. [24] from Sub-Saharan Africa reported adherence to a healthy diet ranging between 33%-87%, while a study by Morge et al. [25] examining adherence in low- and middle-income countries reported 58%. A study by Coyle et al. [11] examining adherence in high-income countries reported healthy diet adherence ranging between 50% and 80.9%. As such, these studies reported a relatively higher prevalence of adherence to dietary habits than those reported in our study. The differences in study outcomes might be due to differences in foods consumed in other countries and the difference in cultural and traditional values. For example, South Asians often eat certain foods because of their cultural and traditional importance, even if they are known to be unhealthy [135]. In addition, South Asians consume high amounts of white rice, other refined grains, saturated fats, and low amounts of fibre and vegetables; these food patterns increase the risk of T2DM [141,142]. Furthermore, poor quality information on dietary modification and misconceptions on what a healthy diet constitutes might have also restricted adherence to healthy dietary behaviours among South Asians [135]. Cost constraints are also a barrier to consuming the recommended amount of fruits and vegetables among South Asians [143]. Evidence shows that the practice of fasting, a religious belief observed with Hindus (Navratri, Mahashivratri, Janmashtami, Ashtami, Ekadashi), Muslim (Ramadan), and Jain (Ekasana, Digambarupvas), has also impacted the practicing of healthy dietary habits [136]. As diet plays an important role in controlling glycaemic levels and preventing T2DM complications [144,145], there is an need for addressing dietary behaviours among people with T2DM in South Asia. This can be achieved by implementing culturally tailored and contextual interventions on healthy diets, given the importance of diet in maintaining the recommended level of blood glucose.

Regular blood glucose monitoring improves blood glucose level among people with T2DM [146] and its frequency varies from person to person depending on the patient’s needs and health care provider’s advice. We found that the pooled prevalence of blood glucose monitoring was 65%. However, studies by Morge et al. [25] (range = 13%-79%), Ketema et al. [22] (pooled prevalence = 28%), and Dagnew et al. [23] (pooled prevalence = 31.89%) found a lower prevalence of blood glucose monitoring than our study. This might be because we only considered those monitoring the blood glucose level at monthly or at least once in three months intervals as adhering to the blood glucose monitoring, while the above-mentioned studies considered daily, weekly, monthly, three-monthly, bi-annually and other intervals. Unlike other self-care behaviours, it was difficult to compare the practice of blood glucose monitoring among people with T2DM due to varied treatment goals. Also, the access to glucometers at the household level in South-Asia is minimal because of cost constraints, thus contributing to suboptimal blood glucose monitoring. The engagement of community health workers in primary health care centres can ensure comprehensive health care services are delivered and self-management of NCDs is promoted [147]. As such, community health workers can be trained to conduct regular visits to patients with T2DM and provide social prescriptions required for adopting healthy self-care behaviour.

The overall pooled prevalence of adherence to foot care was 42%. A lower prevalence of foot care than in this study was observed in the review by Morge et al. [25] which reported a median adherence to foot care of 36.5% (IQR = 13.6%-59.2%). However, the studies conducted by Dagnew et al. [23] (pooled prevalence = 63.61%) and Ketema et al. [22] (pooled prevalence = 58%) reported higher adherence to diabetic foot-care. The lower prevalence of foot care among people with T2DM in the South-Asian region might be the result of the practice of barefoot walking, use of inappropriate footwear, poor awareness of foot care and its complications, and poor counselling on foot care from service providers [148,149]. Foot problems due to T2DM can have a large economic impact which deteriorates the quality of life and ultimately results in physical impairment [150]. There is a high need for health literacy programs on foot care and its complication among people with T2DM in this region.

### Strengths and limitations:

This study has several strengths. This review adhered to the PRISMA guidelines [26] and is registered in the PROSPERO website [151] (Registration number: CRD42021242930). We included cross-sectional and observational studies searching multiple databases and performed forward and backward reference checking to ensure no relevant articles were missed. In addition, this study considered and categorized the studies as those defining the study tool and/or providing a clear definition of self-care domain and those not reporting the study tool and/or not providing a clear definition of self-care domain (reporting only good or poor practice). This allowed for sub-group analyses and pooled prevalence calculation to be done separately. Moreover, this study is the first of its kind to provide comprehensive findings on the practice of self-care behaviours among people with T2DM in South Asian countries.

This study also had some limitations. The findings of the sub-group analysis, specifically among studies not defining study tools and/or not providing a clear definition of self-care domains assessed, should be used with caution. Another limitation was that only “high adherence” to medication intake was categorized as adequate. This is because moderate adherence was unevenly calculated (scoring for moderate adherence differed) among the included studies. In addition, the pooled prevalence of adequate blood glucose monitoring might be over-reported, as we included only those monitoring blood glucose every month or at least once in three months in the meta-analysis. Moreover, the country-specific findings on each domain of self-care should be interpreted with caution, as the number of studies varied between countries, with some countries reporting very low numbers. Finally, outcomes might be biased as most of the included studies only assessed self-reported self-care behaviours.

## CONCLUSION

The findings of this meta-analysis suggest that the overall self-care behaviour among people with T2DM in South Asia was low. Of five self-care domains, blood glucose monitoring and medication adherence were relatively common compared to physical activity, diet, and foot care. There is a need for designing and implementing high-quality, community-based, cost-effective, and culturally-tailored interventions to improve self-care among people with T2DM in South Asia.

## Additional material


Online Supplementary Document
