# Supplementary material for: Self-care behaviours among people with type 2 diabetes mellitus in South Asia: A systematic review and meta-analysis
Source: J Glob Health. 2022 Aug 3;12:04056. doi: 10.7189/jogh.12.04056 (PMC9346342; doi:10.7189/jogh.12.04056)
Supplement: Online Supplementary Document [file jogh-12-04056-s001.pdf]

Appendix S1. List of search terms

"Diabetes Mellitus, Type 2"[Mesh] OR diabetes[tw] OR “anti-diabetic agent”[tw] OR “antidiabetic agent”[tw] OR “hypoglycemic agent”[tw] OR hypoglyc*[tw]
AND
"Self Care"[Mesh] OR self-care[tw] OR “self care”[tw] OR “self-care behavio*”[tw] OR “self care behavio*”[tw] OR self-management[tw] OR self-practice[tw] OR “self-care practice”[tw] OR practice[tw] OR “self care activit*”[tw] OR “self-care activit*”[tw] OR “self-control”[tw] OR “foot care”[tw] OR “dental care”[tw] OR “eye care”[tw] OR smoking[tw] OR “tobacco use”[tw] OR alcohol[tw] OR medication[tw] OR compliance[tw] OR adherence[tw] OR “self-adherence”[tw] OR “treatment adherence”[tw] OR insulin[tw] OR “blood sugar monitor*”[tw] OR “blood sugar test”[tw] OR diet[tw] OR “dietary behavio*”[tw] OR nutrient*[tw] OR nutrition[tw] OR “Food intake”[tw] OR “fast food”[tw] OR exercise[tw] OR “physical activit*[tw] OR walking[tw] OR running[tw] OR swimming[tw] OR “psychosocial support”[tw] OR “family support”[tw] OR “emotional support”[tw] OR “social support”[tw] OR “risk reduction”[tw] OR “health literacy”[tw] OR “health care services”[tw]
AND
"Nepal"[Mesh] OR "South-Asia" OR "India"[Mesh] OR "Pakistan"[Mesh] OR "Bangladesh"[Mesh] OR "Sri Lanka"[Mesh] OR "Bhutan"[Mesh] OR "Indian Ocean Islands"[Mesh] OR "Afghanistan"[Mesh] OR Maldives[tw] OR “Indian subcontinent”[tw]

Appendix S2. Critical appraisal of the included studies

**Quality Assessment**	
High (80% and above)	35 (38%)
Moderate (60-80%)	33 (36%)
Low (<60%)	24 (26%)
